# DC-STAMP knock-down deregulates cytokine production and T-cell stimulatory capacity of LPS-matured dendritic cells

**DOI:** 10.1186/1471-2172-12-57

**Published:** 2011-10-06

**Authors:** Anna Sanecka, Marleen Ansems, Amy C Prosser, Katharina Danielski, Kathrin Warner, Martijn H den Brok, Bastiaan JH Jansen, Dagmar Eleveld-Trancikova, Gosse J Adema

**Affiliations:** 1 Department of Tumor Immunology, Nijmegen Centre for Molecular Life Sciences, Radboud University Nijmegen Medical Centre, Nijmegen, The Netherlands; 2 Lead Pharma Medicine BV, Kapittelweg 29, 6525 EN Nijmegen, The Netherlands

## Abstract

**Background:**

Dendritic cells (DCs) are the highly specialized antigen presenting cells of the immune system that play a key role in regulating immune responses. DCs can efficiently initiate immune responses or induce tolerance. Due to this dual function, DCs are studied in the context of immunotherapy for both cancer and autoimmune diseases. Characterization of DC-specific genes, leading to better understanding of DC immunobiology, will help to guide their use in clinical settings. We previously identified DC-STAMP, a multi-membrane spanning protein preferentially expressed by DCs. DC-STAMP resides in the endoplasmic reticulum (ER) of immature DCs and translocates towards the Golgi compartment upon maturation. In this study we knocked down DC-STAMP in mouse bone marrow-derived DCs (mBMDCs) to determine its function.

**Results:**

We demonstrate that DC-STAMP knock-down mBMDCs secrete less IL-6, IL-12, TNF-α and IL-10 while IL-1 production is enhanced. Moreover, LPS-matured DC-STAMP knock-down mBMDCs show impaired T cell activation potential and induction of Th1 responses in an alloreaction.

**Conclusions:**

We show that DC-STAMP plays an important role in cytokine production by mBMDCs following LPS exposure. Our results reveal a novel function of DC-STAMP in regulating DC-initiated immune responses.

## Background

Dendritic cells (DCs) are professional antigen presenting cells (APC) that play a central role in innate and adaptive immunity. DCs, armed with a wide range of receptors that sense danger signals and scavenge antigens in the surrounding environment, constantly scan our body. Antigen uptake in the presence of inflammation and danger signals results in DC maturation. In this active state DCs are able to efficiently induce immune responses [1]. On the other hand, in the absence of danger signals DCs regulate tolerance to self-antigens in order to prevent autoimmunity.

During maturation DCs upregulate costimulatory molecules such as CD40, CD80 and CD86 as well as MHC class II, which allows for effective antigen presentation to naïve T cells. Furthermore, mature DCs produce and secrete proinflammatory cytokines and chemokines to attract and activate innate effector cells as well as to direct the development of specific T helper (Th) subsets [2]. High levels of IL-12 will induce differentiation of naïve CD4^+ ^T cells into Th1 cells while blocking the development of the Th2 lineage [3]. To prime Th2 responses IL-4 produced by Th2 cells themselves, NKT cells, eosinophils or basophils is needed [4,5]. Additionally, IL-1 has a positive influence on expansion of the murine Th2 cells [6]. The murine Th17 T-cell subset efficiently develops in the presence of the proinflammatory cytokines IL-6 and TGF-β [7].

Due to their immunoregulatory capacities DCs are a promising tool for immunotherapy. Indeed, DC-based therapies are currently being used for treatment of cancer, autoimmune diseases and the prevention of transplant rejection [8-12]. Detailed understanding of molecular aspects of DC immunobiology is crucial for optimal application of DCs in immunotherapy. Characterization of genes like DC-SIGN [13], DC-CK1 [14] and DC-SCRIPT [15-17] has already resulted in many novel findings regarding the molecular basis of DC function.

Recently, we reported on the isolation and characterization of a novel molecule named DC-STAMP, both in human and mouse DCs [18,19]. DC-STAMP was shown to be a multi-membrane spanning protein preferentially expressed by myeloid DCs [18], macrophages [20] and osteoclasts [21]. In immature DCs, DC-STAMP localizes to the endoplasmic reticulum [22] and upon DC maturation translocates towards the Golgi compartment, which is most likely facilitated by its interacting partner OS9 [23], a protein that has previously been implicated in ER-to-Golgi transport [24,25]. Interestingly, DC-STAMP also interacts with the ER-resident transcription factor LUMAN [26]. LUMAN is activated in a process called regulated intramembrane proteolysis (RIP), which involves its translocation to the Golgi compartment, proteolytic cleavage and subsequent nuclear localization [27]. The immunological and biological processes DC-STAMP is involved in are only recently emerging. Functional studies in DC-STAMP knock-out mice have shown that DC-STAMP is essential for fusion of osteoclasts and foreign body giant cells [21,28]. Much less is known regarding the role of DC-STAMP in myeloid immune cells. DC-STAMP was shown to inhibit granulocyte development from hematopoietic progenitors cells [29], however its expression is not required for proliferation and differentiation of DCs [30]. Initial data using immature DCs from DC-STAMP knock-out mice have suggested involvement of DC-STAMP in phagocytosis and antigen presentation. As aged DC-STAMP knock-out mice show symptoms of autoimmune diseases, a role of DC-STAMP in maintaining the balance between immunity and tolerance has been proposed [30].

In the current study we examined the role of DC-STAMP in immature and TLR-matured DCs. For this purpose, we generated lentiviruses encoding DC-STAMP-specific shRNAs to knock-down DC-STAMP in BMDCs. We found that DC-STAMP knock-down in mature but not immature DCs affects cytokine production, induction of T cell proliferation and Th1 cell activation.

## Results

### DC-STAMP silencing in mouse bone marrow-derived DCs

**Figure 1:**
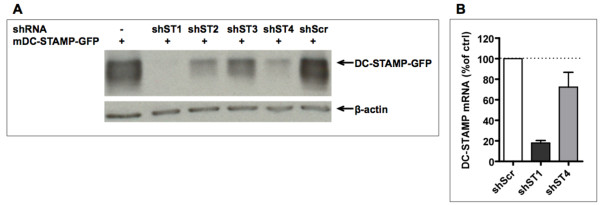
**Efficiency of DC-STAMP silencing**. (A) HEK293 cells were co-transfected with mDC-STAMP-GFP plasmid and one of four plasmids expressing different shRNA sequences, designed to silence DC-STAMP, or with plasmid expressing scrambled shRNA. Cells were lysed 48 hours post-transfection and lysates were subjected to Western blot analysis. Blots were stained for GFP and re-probed for β-actin as an internal reference. Data shown are representative of two independent experiments. (B) Murine BMDCs were transduced with lentivirus expressing shST1 or shST4 to silence DC-STAMP, or shScr as a negative control. Messenger RNA expression of DC-STAMP was assessed by qPCR. Levels of normalized DC-STAMP mRNA expression in cells infected with shST1 and shST4 lentiviruses were related to normalized DC-STAMP mRNA expression in shScr lentivirus infected cells. Data are depicted as mean percentage of control (shScr) ± SEM of three independent experiments.

To investigate the role of DC-STAMP in DCs, we performed DC-STAMP knock-down studies in mBMDCs. Hereto, four different DC-STAMP shRNA sequences and a control scrambled shRNA sequence (shScr) were tested for their ability to silence murine DC-STAMP-GFP following co-transfection in HEK293 cells. Silencing was assessed by western blot analysis using antibodies directed against the GFP-moiety of the DC-STAMP-GFP fusion protein (Figure 1A). The results show that the shRNA sequences shST1 and shST4 were most effective in DC-STAMP silencing whilst the scrambled shRNA had no effect. Therefore, these two DC-STAMP shRNA sequences were chosen for further use in mBMDCs. As mBMDCs are difficult to transfect, we used lentiviral shRNA delivery to stably produce shRNA from an integrated RNAi cassette and avoid as much as possible the host's defense mechanisms. Assessment of the effect of lentiviral transduction on maturation marker expression and cytokine production at the time of DC stimulation, revealed comparable levels of CD86 and MHC class II expression between non-transduced and mock transduced (shScr) mBMDCs. Additionally, proinflammatory and anti-inflammatory cytokines were not detectable in the supernatants of both non-infected and infected cells (data not shown). Taken together, these results indicate that infected cells did not mature spontaneously.

Bone marrow-derived DCs were infected with lentivirus expressing shST1, shST4 or shScr at day 6 and day 7 of culture. As specific antibodies against mDC-STAMP are not available, silencing of mDC-STAMP in mBMDCs was determined at the mRNA level by quantitative real-time PCR (qPCR). Effective silencing of DC-STAMP mRNA was observed at both day 9 and day 11 of culture (40 and 90 hours after second transduction) (Figure 1B and data not shown). DCs at day 11 of culture were used for further experiments. In accordance with the results in HEK293 cells, shST1 was more effective in silencing DC-STAMP (70% silencing) as compared to shST4 Collectively, these data show that we are able to effectively silence DC-STAMP mRNA in mBMDCs with two independent shRNAs.

### DC-STAMP silencing does not induce phenotypic changes in mBMDCs

**Figure 2:**
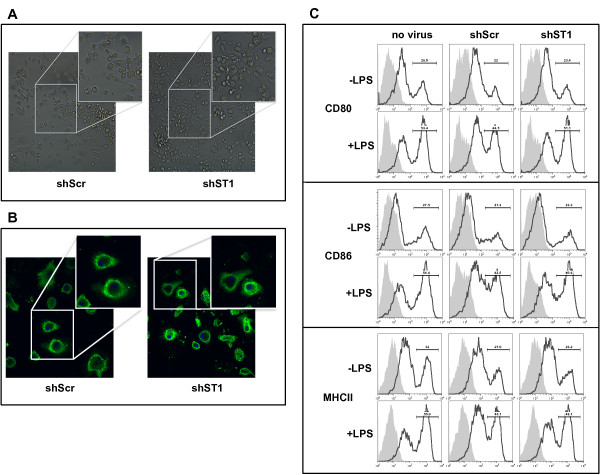
**DC-STAMP silencing does not induce phenotypic changes in mBMDCs**. Murine BMDCs were infected with shScr and shST1 lentivirus at day 6 and day 7 of culture. (A) Light field microscopy image of mBMDCs in culture 4 days post-infection. (B) Lentiviraly transduced mBMDCs stained with antibody against calreticulin (green) to visualize ER and DAPI staining of nucleus (blue) analyzed by CLSM. (C) Flow cytometric analysis of CD80, CD86 and MHC class II surface expression in CD11c^+ ^immature (-LPS) and LPS-matured (+LPS) non-infected (no virus), shScr and shST1 mBMDCs. Isotype controls are indicated by shaded area. Data shown are representative of two or three independent experiments.

First, the effect of DC-STAMP silencing on DC morphology and appearance was determined. Observation of the cells in culture after infection (day 6 and 7) until day 11 of culture did not reveal significant differences in DC morphology of both the non-adherent and loosely adherent cells between DC-STAMP knock-down and control cells (Figure 2A). As DC-STAMP is a molecule residing in the endoplasmic reticulum, we investigated the ER in DC-STAMP knock-down and control cells in more detail by staining for the ER-residing protein calreticulin. Confocal microscopy analysis of the ER did not show any substantial differences in ER abundance or morphology in the DC-STAMP knock-down cells (Figure 2B). This confirms results from DC-STAMP deficient mice indicating that DC-STAMP does not affect the appearance of the ER in DCs [30].

To evaluate the effects of DC-STAMP silencing on the expression of DC surface molecules flow cytometry analysis was performed. Expression of MHC class I and class II, and the costimulatory molecules, CD80, CD86 and CD40 was analyzed on immature and LPS-matured DC-STAMP knock-down (shST1 and shST4) and control (shScr) mBMDCs. To monitor the effect of viral infection, non-infected cells (no virus) were taken along in this analysis. No significant differences between control (shScr) and DC-STAMP knock-down immature mBMDCs were observed for any of the surface markers analyzed (Figure 2C and data not shown). Upon LPS stimulation for 24 hours, CD80, CD86, and MHC class II expression levels were elevated in both control and DC-STAMP-silenced DCs (Figure 2C and data not shown). Non-infected and control shRNA (shScr) infected mBMDC did not significantly differ in their response to LPS stimulation, although the upregulation of costimulatory molecules in lentivirus infected cells was somewhat less. Furthermore, no differences in overall morphology or cell death were observed between the DC-STAMP knock-down and control cells following LPS-induced DC maturation. These data show that the phenotype of DC-STAMP knock-down cells does not differ from the control cells and correlates with results obtained from DC-STAMP knock-out mice [30]. Additionally, our data show that the upregulation of costimulatory molecules following maturation is not affected in DC-STAMP silenced DCs relative to control cells.

### LPS-induced cytokine secretion is altered in DC-STAMP knock-down mBMDCs

**Figure 3:**
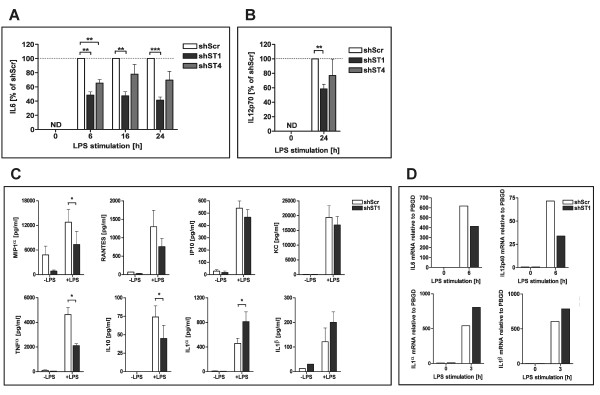
**LPS-induced cytokine secretion is altered in DC-STAMP knock-down mBMDCs**. Murine BMDCs transduced with shST1, shST4 or shScr were stimulated with 1 μg/ml of LPS for 6, 16 and 24 hours. (A) Levels of secreted IL-6 were measured by ELISA. Data are expressed as a percentage of control (shScr). Mean ± SEM calculated of four independent experiments. Values in all experiments ranged from 5 to 500 ng/ml. (B) Levels of secreted IL-12 p70 were measured by ELISA. Data shown are representative of three independent experiments and are expressed as a percentage of control (shScr). Mean ± SEM calculated from triplicates. Values in all experiments ranged from 0.4 to 5 ng/ml. (C) Levels of secreted cytokines were determined in supernatants 0 and 24 hours after LPS stimulation using Milliplex bead assay. Mean ± SEM calculated from at least three independent experiments. Two-tailed Student *t *test was performed in A, B and C (ND-not detectable, *p < 0.05; **p < 0.01; ***p < 0.001). (D) Total RNA was isolated from shScr and shST1 mBMDCs stimulated with LPS. Messenger RNA expression levels of IL-6, IL-12p40, IL-1α and IL-1β were determined by quantitative RT-PCR where PBGD served as an internal reference. Results shown are representative of at least three independent experiments. The differences in mRNA levels between shScr and shST1 in all experiments ranged from 20% to 75% for IL-6, 20-65% for IL-12p40, 25-48% for IL-1α and 27-35% for IL-1β.

Upon DC maturation, along with changes in cell surface molecule expression, DCs also begin secreting cytokines and chemokines that play an important role in regulating immune responses. To determine the capacity of DC-STAMP knock-down mBMDCs to produce cytokines, cells were either left unstimulated or stimulated with LPS for 6, 16 and 24 hours and the production of IL-6 was measured by ELISA. No difference in the cytokine production by the mBMDCs was observed without LPS stimulation, indicating that knock-down of DC-STAMP does not induce spontaneous IL-6 production. In contrast, the amount of IL-6 in supernatants of the DC-STAMP knock-down DCs after LPS stimulation was significantly decreased relative to control mBMDCs at all time points (Figure 3A). Moreover, this effect was proportional to the level of DC-STAMP silencing. Cells transduced with shST1 produced 50% less of IL-6 than control mBMDCs whilst cells transduced with shST4 produced only 20-30% less. Next, IL-12 p70 levels were measured in supernatants of DC-STAMP knock-down and control DCs. No IL-12 was spontaneously induced in immature DC-STAMP knock-down DCs (Figure 3B). Interestingly, IL-12 p70 was readily detected at 16 and 24 hours of LPS stimulation, but the levels were significantly lower in DC-STAMP knock-down mBMDCs (shST1) (Figure 3B). The finding that the level of DC-STAMP down-regulation with the two independent shST1 and shST4 DC-STAMP shRNAs is proportional to the decrease in IL-12 production further demonstrates the relevance of this observation.

To further define the effect of DC-STAMP on cytokine and chemokine production, supernatants of immature and 24 hours LPS-matured mBMDCs transduced with shST1 and shScr were analyzed by Milliplex bead assay. As expected, the predominantly T cell derived cytokines IFN-γ, IL-2, IL-4, IL-5, IL-9, IL-17, as well as IL-7 and IL-15 were not present at detectable levels in the supernatants of mBMDCs irrespective of DC-STAMP silencing and LPS stimulation (data not shown). Expression of the chemokines RANTES, IP-10, KC, MCP-1, MIP-2 as well as growth factor VEGF was readily detected upon LPS stimulation but no significant differences between shST1 and shScr control cells were observed (Figure 3C and data not shown). In contrast, the proinflammatory chemokine MIP-1α was expressed by both immature and mature DC and was clearly decreased in the supernatants of both immature and LPS-matured DC-STAMP knock-down DCs (Figure 3C). Secretion of TNF-α and the anti-inflammatory cytokine IL-10 was also significantly decreased in supernatants of the LPS-matured DC-STAMP knock-down mBMDCs. On the other hand, LPS induction of the proinflammatory cytokine IL-1α was significantly increased in DC-STAMP knock-down mBMDCs relative to control DCs at the 24 hour time point. IL-1β levels were also increased in supernatants of mature DC-STAMP knock-down mBMDCs although the difference did not reach statistical significance (Figure 3C). Further analysis revealed that the increase in IL-1α production in LPS-matured DC-STAMP silenced DCs is not present at earlier time points (6 and 16 hours) (data not shown).

To address whether the observed deregulation in cytokine production can be explained by an effect of DC-STAMP silencing on cytokine transcription, the levels of the IL-6, IL-12p40, IL-1α and IL-1β mRNA were assessed (Figure 3D). Detected differences in mRNA levels fluctuated between experiments, however the same tendency was observed. IL-6 and IL-12p40 mRNA levels were decreased in DC-STAMP knock-down cells (shST1) (20-75% for IL-6, 20-65% for IL-12p40), while mRNA of IL-1α and IL-1β levels were increased (25-48% for IL-1α and 27-35% for IL-1β).

Taken together, these results demonstrate that DC-STAMP deficiency results in the deregulation of cytokine production by mBMDCs and suggest the involvement of transcriptional regulation of cytokine genes in this phenomenon.

### DC-STAMP deficiency leads to decreased T-cell proliferation

**Figure 4:**
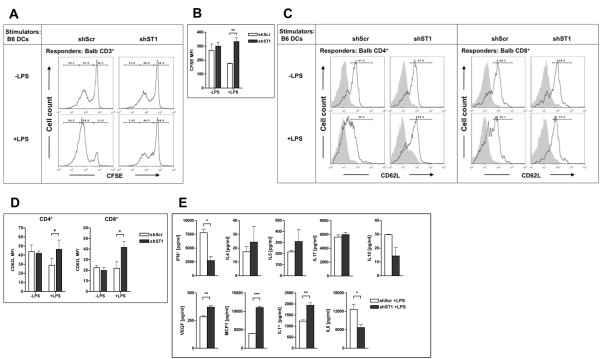
**Decreased ability of DC-STAMP knock-down mBMDCs to stimulate T cell proliferation in allogeneic MLR**. Splenocytes depleted for B220^+ ^cells from Balb/c mice were stained with CFSE and co-cultured with immature (-LPS) or LPS-matured (+LPS) DC-STAMP knock-down (shST1) or control (shScr) mBMDCs from C57BL/6 mice. (A) FACS data of CFSE dilution in CD3^+ ^T cells. (B) Quantified T-cell proliferation as a mean fluorescent intensity (MFI) of CFSE dilution in CD3^+ ^T cells. Mean ± SEM of four independent experiments are shown. (C) FACS data of CD62L surface expression in CD3^+^CD4^+ ^and CD3^+^CD8^+ ^T cells (Balb/c) from co-cultures with DC-STAMP knock-down (shST1) or with control (shScr) mBMDCs (C57BL/6) (black line) compared to isotype control (grey shaded area). Data shown are representative of three independent experiments. (D) Quantification of CD62L MFI on CD3^+^CD4^+ ^and CD3^+^CD8^+ ^T cells (Balb/c) from co-cultures with DC-STAMP knock-down mBMDCs (shST1) and shScr mBMDCs (C57BL/6). Data are expressed as mean ± SEM of three independent experiments. (E) Supernatants from allogeneic MLR co-cultures with mature mBMDCs were analyzed for indicated cytokines and chemokines by Milliplex bead assay. Data shown are mean ± SEM from triplicates and are representative of at least three experiments. Two-tailed Student *t *test was performed in B, D and E (ND-not detectable, *p < 0.05; **p < 0.01; ***p < 0.001).

Next, we investigated the capacity of immature and LPS-matured DC-STAMP knock-down and control DCs to stimulate proliferation of CFSE-labeled allogeneic splenocytes depleted for B220 positive cells. The proliferation rate of total CD3^+ ^T cells co-cultured with immature mBMDCs did not differ between DC-STAMP knock-down and control cells (Figure 4A and 4B). In contrast, when the LPS-matured DC-STAMP knock-down mBMDCs were used as stimulators a significantly decreased proliferation rate of total CD3^+ ^T cells was observed (Figure 4A and 4B). In line with this finding, a significantly higher expression of CD62L was still present on CD4^+ ^as well as CD8^+ ^T cells in the cultures stimulated with the matured DC-STAMP knock-down mBMDCs compared to those stimulated with control DCs (Figure 4C and 4D). No difference in CD62L expression was observed on the T cells activated by the immature mBMDCs. These data confirm that DC-STAMP knock-down mature mBMDCs have an impaired ability to activate T cells in an allogeneic MLR.

T cells stimulated by activated DCs can differentiate into different directions to promote Th1, Th2 or Th17 type responses. As we observed differences in proliferation rate and activation of T cells of co-cultures stimulated with mature mBMDCs, we analyzed supernatants from these co-cultures for the presence of a number of cytokines and chemokines (Figure 4E). Strikingly, the levels of IFN-γ, a cytokine produced mainly by CD4^+ ^cells of the Th1 phenotype, were significantly reduced in supernatants from co-cultures with DC-STAMP knock-down mature mBMDCs. Cytokines produced by Th2 type cells, IL-4 and IL-5, were present in somewhat higher amount in supernatants of co-cultures with DC-STAMP knock-down mBMDCs although these differences were not statistically significant. No difference in IL-17 levels was observed in the co-cultures with DC-STAMP knock-down and control mBMDCs while IL-10 was decreased without reaching statistical significance. Corresponding to our results obtained from supernatants of mBMDCs cultures, IL-6 expression was decreased and IL-1α expression was increased in co-cultures with mature DC-STAMP knock-down mBMDCs. The proinflammatory chemokines MCP-1 and VEGF were present in significantly higher quantities in supernatants of co-cultures with DC-STAMP knock-down mBMDCs (Figure 4E) relative to control DC. Together, these results show that LPS-matured DC-STAMP knock-down mBMDCs are less efficient in stimulating T cell proliferation and initiating IFN-γ producing Th1 cells.

## Discussion

DCs are professional antigen-presenting cells that are critically involved in the initiation of the primary immune response [1]. Maturation of the DC is important for priming naïve T cells and the generation of appropriate T cell responses. Here we show that silencing of the DC-expressed molecule DC-STAMP results in a distorted cytokine production both on mRNA and protein level in mBMDCs following LPS exposure. The importance of DC-STAMP in mBMDC maturation is further emphasized by the decreased ability of mature but not immature DC-STAMP knock-down DCs to stimulate naïve T cells and to prime Th1 responses.

Studies of DC-STAMP overexpression in murine bone marrow progenitor cells have suggested that DC-STAMP affects granulocyte development from hematopoietic progenitor cells [29]. On the other hand, analysis of DC-STAMP knock-out mice mBMDCs shows that DC-STAMP is not necessary for DC proliferation and differentiation [30]. Little is known about the role of DC-STAMP in the maturation of DCs. DC maturation is a key checkpoint in the initiation of immunity and has important consequences on the quality of the immune response. Our previous studies have shown translocation of DC-STAMP from ER towards the Golgi compartment upon maturation of DCs [23]. This differential localization of DC-STAMP in immature and mature DCs may suggest that DC-STAMP exerts an important role during the complex process of maturation. No significant differences in the morphology and the expression of DC-maturation markers between DC-STAMP knock-down and control immature and mature mBMDCs was observed. These data indicate that DC-STAMP silencing does not affect the phenotype of mBMDCs.

Upon TLR ligation, besides co-stimulatory molecule expression, DCs also start to produce a wide range of cytokines and chemokines to attract and stimulate other cells of the immune system [1]. Our data show that despite the mature cell surface phenotype, DC-STAMP knock-down mBMDCs exhibit distorted cytokine production upon LPS stimulation (Figure 3). Further analysis indicated that observed differences in secretion of IL-6, IL-12, IL-1α and IL-1β proteins are accompanied by the corresponding changes in mRNA levels (Figure 3D), suggesting regulation at the transcriptional level. Interestingly, one of the DC-STAMP interacting partners, LUMAN, is a transcription factor that resides in the ER. For its activation LUMAN is translocated towards the Golgi compartment, cleaved and subsequently accumulates in the nucleus [27]. Previously, we have shown that LUMAN protein in human myeloid DCs becomes translocated to the nucleus following TLR4 ligation in mature DCs [26]. Others have shown that LUMAN can bind to cAMP response element (CRE) and unfolded protein response element (UPRE) transcription factor binding sites in promoters of target genes [31-33]. CRE sites are present in promoters of many cytokines, including, IL-2, IL-6, IL-10 and TNF-α [34]. It is therefore tempting to speculate that LUMAN is responsible at least in part for the deregulation of cytokine production observed in TLR-matured DC-STAMP knock-down DCs. LUMAN knock-down studies are required to confirm this hypothesis.

We further observed that levels of IL-1α and IL-1β were increased in DC-STAMP knock-down mBMDCs. This upregulation of IL-1 family cytokines seems to be contradictory to the observed decrease in IL-6 production as it is well established that IL-1 is able to induce expression of IL-6 [35,36]. The fact that IL-6 is also implicated in inhibition of IL-1 and TNF-α production [37,38], however, indicates that the relation between IL-6 and IL-1α and IL-1β is more complex. Looking at the mRNA level, IL-6 and IL-1 mRNA production is primarily affected by decreased DC-STAMP expression at early time points. The decrease in IL-6 protein level was observed early on after LPS stimulation, while translation and release of IL-1α and IL-1β took much longer. These data suggest that the cytokine profile in DC-STAMP KO DC may be explained by differences in the kinetics in the transcription, translation and release of IL-6 and IL-1 cytokines, and could possibly occur through the involvement of caspase-1/calpain in IL-1β/IL-1α release [39,40].

Many factors influence the differentiation process of CD4^+ ^T cells into Th1, Th2 or Th17 effector cells, including antigen dose, the signal strength through the T cell receptor, and costimulation [41]. Especially cytokines were shown to be key determinants in the outcome of this differentiation [2-7]. In our MLR assays we observed negative effect of DC-STAMP silencing on T cell activation by mature DCs. Decreased levels of IFN-γ in these co-cultures, indicate an impaired ability of mature DC-STAMP knock-down mBMDCs to prime Th1 responses. As IL-12 is critically involved in the promotion of Th1 development [3], we postulate that this decreased ability to support the development of Th1 lineage is determined by the reduced production of IL-12 by LPS-stimulated DC-STAMP knock-down mBMDCs. This is also supported by the fact that expression of costimulatory molecules was not affected by DC-STAMP knock-down. Impaired stimulation of Th1 responses allows differentiation of Th2 and Th17 subsets. Additionally, IL-1α and IL-1β were shown to stimulate Th2 and Th17 responses [6], as well as induce the release of Th2 cytokines [42]. The observed increase in IL-4 and IL-5 levels in co-cultures with mature DC-STAMP knock-down mBMDCs could suggest skewing the responses towards Th2 cells, which may be enhanced due to the higher levels of IL-1 cytokines in these co-cultures. Lower number of CD8^+ ^T cells alone was observed after co-culture with DC-STAMP knock-down mature mBMDCs, however cells, which were dividing, went through the same number of divisions like the ones in co-culture with control mBMDC (data not shown). We postulate that the decrease in the CD8^+ ^T cells proliferation is a result of the reduced CD4^+ ^T cells help.

Sawatani *et al*. showed that aged DC-STAMP knock-out mice have symptoms of autoimmune disease and suggested involvement of increased phagocytosis and antigen presentation in onset of autoimmunity [30]. We did not observe any differences in T cell proliferation between control and DC-STAMP knock-down cells when we used immature DC. This discrepancy between our and Sawatani *et al*. data may be explained by different design of the proliferation assays. We used allogeneic MLR to look at the ability of the DC-STAMP knock-down mBMDCs to stimulate differentiation of naïve T cells while they looked at antigen-specific (OVA) responses. Decreased levels of proinflammatory cytokines IL-6, IL-12 and TNF-α do not easily explain the symptoms of autoimmune diseases observed in DC-STAMP-deficient mice. On the other hand, diminished production of anti-inflammatory cytokine IL-10 could be dominant over the reduction in proinflammatory cytokines. As IL-10 is crucially involved in preventing excessive immune responses [43] and plays an important role in development of suppressor T cells [44,45] decrease in its production could lead to autoimmune disease.

## Conclusions

In conclusion we clearly show the importance of DC-STAMP expression in cytokines production by mature mBMDCs. Further studies are necessary to resolve the pathway by which this phenomenon occurs. We postulate that the deregulated cytokines production in DC-STAMP knock-down mBMDCs upon LPS stimulation is responsible for impaired T-cell stimulatory capacity of these cells.

## Methods

### Cell lines and cell culture

The human embryonic kidney cell line HEK293 was cultured in DMEM (Invitrogen) supplemented with 10% heat-inactivated FCS (Greiner Bio-One), 1% of non-essential amino acids (NEAA) (Invitrogen) and 0.5% antibiotic-antimycotic (Invitrogen). The mouse embryonic fibroblast cell line NIH3T3 was cultured in DMEM supplemented with 5% heat-inactivated FCS, and 0.5% antibiotic-antimycotic. HEK293FT cells (Invitrogen) were cultured in DMEM supplemented with 10% heat-inactivated FCS, 1% NEAA, 0.5% antibiotic-antimycotic, 1% ultra-glutamine (Lonza), and 1 mM sodium pyruvate (Invitrogen). Cells were kept under selection with 500 μg/ml of Geneticin (G418) (Invitrogen).

### Generation of bone marrow-derived DCs

Mouse BMDCs were generated from bone marrow progenitor cells, according to the modified protocol of Lutz [46]. Bone marrow cells were flushed from the femurs and tibias of 6- to 8-week-old female C57BL/6 mice (Charles River WIGA Gmbh), washed and counted. Cells were plated at a concentration of 4 × 106 cells per 10 cm Petri dish in 13 ml of RPMI-1640 medium (Invitrogen) supplemented with 10% FCS, 1% ultra-glutamine, 28 μM of β-mercaptoethanol (Sigma-Aldrich), 0.5% antibiotic-antimycotic and 20 ng/ml of murine recombinant GM-CSF (PeproTech). After 3 days, 4 ml of fresh medium was added containing fresh GM-CSF to final concentration of 8.75 ng/ml. At day 6 non-adherent and loosely adherent cells were harvested and used for transduction.

### Vectors and validation of RNAi

The construction of murine DC-STAMP-GFP vector was described previously [22]. Four SureSilencing shRNA plasmids encoding the shRNA sequence targeting murine DC-STAMP: shST1 (5'-gctggaagttcacttgaaact-3'), shST2 (5'-ttgtggctggaagtatgagaatgt-3'), shST3 (5'-tctggatgatcacctgtgttt-3'), shST4 (5'-ggttcctctcagtattattct-3') and negative control Scrambled shRNA (shScr) (5'-ggaatctcattcgatgcatac-3') plasmid were obtained from SuperArray Bioscience. In order to validate the silencing of mDC-STAMP, HEK293 cells were co-transfected with a plasmid expressing mDC-STAMP-GFP plasmid and a SureSilencing shRNA plasmid encoding either a control shRNA or mDC-STAMP-targeting shRNA using Lipofectamine (Invitrogen) according to the manufacturer's protocol. Forty-eight hours following transfection, mDC-STAMP-GFP protein levels were determined by Western blot.

### Western blotting

Cells were lysed in 1% SDS buffer and subjected to Western blot analysis as described previously [22]. GFP-tagged mDC-STAMP was detected using mouse anti-GFP antibody (Roche). Actin was detected with a mouse anti-β-actin Ab (Sigma-Aldrich). Rabbit anti-mouse-HRP (DakoCytomation) was used as a secondary antibody. For detection an ECL Western Blotting Detection Reagents kit (Amersham Bioscience) and BioMAx XAR Film (Kodak) were used.

### Generation of the lentiviral vector stocks

Using Gateway technology, DNA encoding sequences for short-hairpin RNAs in the BLOCK-iT-U6-RNAi Entry vector were introduced into the pLenti6/BLOCK-iT-DEST vector (Invitrogen), following the manufacturer's protocol. The individual vectors were co-transfected with packaging vectors into the HEK293FT cells according to the manufacturer's instructions. Virus was produced in HEK293FT culture medium supplemented with 82.5 μg/ml of water-soluble cholesterol (Sigma-Aldrich) and collected from tissue culture supernatant 24 and 48 hours after transfection. Cell debris was removed by centrifugation and supernatant containing virus particles was filtered through a 0.45 μm filter. Aliquots were stored at -80°C until use.

Viral titers were determined by transduction of NIH3T3 cells with serial dilutions of the virus stock in the presence of 10 μg/ml of polybrane (Sigma-Aldrich). Two days after transduction the cells were cultured under selection with 2 μg/ml of Blasticidin (Invitrogen) for 12 days. Medium was refreshed every third day. To determine the titer, the number of colonies was determined after crystal violet staining. The titer ranged between 2 × 105 to 13 × 105 transfection units (TU)/ml

### Lentiviral knockdown of mDC-STAMP in mBMDCs

Mouse BMDCs were harvested after 6 days of culture and 1.2 × 106 cells were resuspended in 700 ul of virus supernatant containing 10 μg/ml of DEAE-dextran (Pharmacia Biotech AB) to facilitate viral infection. Cell-virus suspensions were plated on 12-well tissue culture plates (700 μl/well) and centrifuged for 90 minutes at 2200 rpm and 37°C. After centrifugation, virus supernatant was replaced by mBMDC culture medium. The infection procedure was repeated the following day. As a negative control, mBMDC culture medium with DEAE-dextran was used. The multiplicity of infection (MOI) for silencing of DC-STAMP was matched with the MOI of the scrambled shRNA control in each experiment.

### RNA isolation and quantitative real-time PCR (qPCR)

Total RNA was isolated from at least 6 × 105 cells using a Quick-RNA MiniPrep kit (Zymo Research). RNA quantity and purity were determined using a NanoDrop spectrophotometer. Two micrograms of total RNA were treated with DNase-I (Invitrogen) and cDNA was synthesized using random primers and SuperScript Moloney murine leukemia virus reverse transcriptase (II-MMLV) (Invitrogen). Messenger RNA levels for the genes of interest were determined with a Bio-rad CFX96 (Bio-rad) using Fast Start SYBR Green kit (Roche) and primers for one of the following genes: porphobilinogen deaminase (PBGD) (forward: 5'-CCTACCATACTACCTCCTGGCTTTAC-3'; reverse: 5'-TTTGGGTGAAAGACAACAGCAT-3'), DC-STAMP (forward: 5'-TTGCCGCTGTGGACTATCTG-3'; reverse: 5'-GAATGCAGCTCGGTTCAAAC-3'), IL-6 (forward: 5'-TGGGAAATCGTGGAAATGAG-3'; reverse: 5'-CAAGTGCATCATCGTTGTTC-3'), IL-1α (forward: 5'-CGAAGACTACAGTTCTGCCATT-3'; reverse: 5'-GACGTTTCAGAGGTTCTCAGAG-3'), IL-1β (forward 5'-GTGATGAGAATGACCTGTTCTTTG-3'; reverse: 5'-GATTTGAAGCTGGATGCTCTC-3'), IL-12p40 (forward 5'-GACACGCCTGAAGAAGATGAC-3'; reverse: 5'-TAGTCCCTTTGGTCCAGTGTG-3'). Data were analyzed with Bio-rad CFX manager version 1.6 (Bio-rad) and checked for correct amplification and dissociation of the products. PBGD served as a reference gene. DC-STAMP and IL-6 levels relative to PBGD were calculated as: 2^-(ΔCt).

### Cytokine measurements

For cytokine assays, 1 × 105 day 11 mBMDCs were plated per well on 96-well plate in 200 μl of culture medium. Cells were stimulated with 1 μg/ml of LPS (E. coli, 0111:B4, Sigma-Aldrich). Supernatants were harvested at the indicated time points and stored at -80°C. Concentrations of IL-12p70 and IL-6 in cell culture supernatants were measured by ELISA (BD Biosciences), according to manufacturer's protocol.

The cytokines and chemokines IL-10, IFN-γ, IL-15, IL-17, IL-1α, IL-1β, IL-2, IL-4, IL-5, IL-7, IL-9, IP-10 (CXCL10), KC (CXCL1), MCP-1 (CCL2), MIP-1α (CCL3), MIP-2 (CXCL2), RANTES (CCL5), TNF-α and VEGF in cell culture supernatants were measured using the mouse cytokine multiplex (Milliplex, Millipore) following the manufacturer's protocol. Data analysis was performed using Bio-Plex Manager software (Bio-Rad Laboratories).

### Immunofluorescent staining and Confocal Laser Scanning Microscopy

Immature mBMDCs (day 11) were seeded onto cover slides coated with Poly-L-Lysine (Sigma-Aldrich) (5 × 105 cells/slide), adhered for 2 hours and fixed with 1% PFA for 15 minutes. Cells were permeabilized with methanol (-20°C; 1 min) and blocked with 3% BSA (Calbiochem) in 1 × PBS supplemented with 0.1% saponin (Sigma-Aldrich). Rabbit anti-calreticulin (Calbiochem) antibody was used to visualize ER. As an isotype control, purified rabbit IgG (Sigma-Aldrich) was used. As a secondary antibody, goat anti-rabbit-Alexa488 (BD Biosciences) was used. The nucleus was stained with DAPI. Slides were mounted in Mowiol (Calbiochem) and analyzed by CLSM using an Olympus FV1000.

### Mixed lymphocyte reactions

Mixed lymphocyte reactions (MLRs) were performed using day 12 C57BL/6 mBMDCs as stimulators. Splenocytes from Balb/c mice (Harlan) depleted for B220- positive cells were used as responders. Negative selection for B220-positive cells was performed to increase T cell numbers in the splenocyte pool. Briefly, splenocytes were stained with an anti-B220-FITC antibody (BioLegend) then incubated with magnetic beads coupled to anti-FITC antibodies and purified with a magnetic bead-based kit (MACS; Miltenyi-Biotec). Cells were labeled with 1 μM CFSE (Invitrogen) at a concentration of 50 × 106 B220-negative cells/ml for 7 min at 37°C in PBS with 1% FCS. MLR assays were carried out in 96-well round-bottom plates (200 μl/well) at a ratio of 1:2 or 1:3 (responders to stimulators), for 4 to 5 days in T cell medium (IMDM (Invitrogen) containing 10% FCS, 1% antibiotic-antimycotic, 0.5% ultra-glutamine, 28 μM of β-mercaptoethanol and 60 IU of human IL-2 (Proleukin; Chiron BV, Amsterdam, The Netherlands). The proliferation rate was assessed by FACS analysis of CFSE dilution.

### Flow cytometry

For cell surface labeling, the following anti-mouse antibodies were used (from BD Biosciences, unless stated differently): PE-conjugated anti-CD86 (GL1), anti-CD80 (16-10A1), anti-CD40 (3/23), anti-CD8α (53-6.7), anti-MHC class II (M5/114.15.2, eBioscience); PerCP-conjugated anti-CD62L (Mel-14, BioLegend); APC-conjugated anti-CD11c (N418, BioLegend); APCCy7-conjugated anti-CD4 (L3T4). Flow cytometry was performed using a CyAn flow cytometer (Beckman Coulter) and analyzed with FlowJo software (TreeStar).

### Statistical analysis

Statistical significance was calculated using a two-tailed Student t test (GraphPad Prism version 4.00 software). Data obtained from ELISA and Milliplex experiments were log2-transformed before statistical analysis. Significance of difference was determined by the p value (*p < 0.05; **p < 0.01; ***p < 0.001).

## Abbreviations

**APC: **antigen presenting cell; **CRE: **cAMP response element; **DC: **dendritic cell; **ER: **endoplasmic reticulum; **mBMDC: **mouse bone marrow-derived dendritic cell; **MFI: **mean fluorescence intensity; **MLR: **Mixed lymphocyte reaction; **ND: **not detectable; **PBGD: **porphobilinogen deaminase; **qPCR: **quantitative PCR; **UPRE: **unfolded protein response element.

## Authors' contributions

AS carried out most experiments and wrote the original manuscript; MA secured mouse bone marrow and co-optimized transduction technology; AP optimized the MLR assay, produced the virus, performed ELISA and FACS experiments; KD determined silencing efficiency of DC-STAMP by different shRNA; KW optimized lentiviral production and transduction of mBMDCs; M den B and BJJ helped with set up of the experiments and statistical analysis; DE-T designed and supervised silencing studies and performed confocal microscopy; GJA supervised the studies and wrote the manuscript. All authors read and approved the final manuscript.
